# A new journal devoted to patient safety in surgery: the time is now!

**DOI:** 10.1186/1754-9493-1-1

**Published:** 2007-11-07

**Authors:** Philip F Stahel, Pierre-Alain Clavien, Dieter Hahnloser, Wade R Smith

**Affiliations:** 1 Department of Orthopaedic Surgery, Denver Health Medical Center, University of Colorado School of Medicine, 777 Bannock Street, Denver, CO 80204, USA.; 2 Department of Visceral Surgery, University Hospital Zurich, Ramistr. 100, Zurich, CH-8091, Switzerland.

## 

For patients, surgical complications are analogous to "friendly fire" in wartime. Both scenarios imply that harm is unintentionally done by somebody whose aim was to help. One would assume that any patient admitted to a hospital to undergo a surgical procedure should expect to be better off after the intervention than before. However, while we, as surgeons, strive to achieve excellent results and ideal patient outcomes, we fail this noble task more often than we appreciate [1]. Interestingly, adverse events resulting from surgical interventions are more frequently related to mistakes and failures before and after surgery than during the operative procedure itself [2]. A recently published analysis of the American College of Surgeons' closed claims study revealed that at total of 97% of all events leading to medicolegal claims involved a delay in diagnosis, a failure to diagnose, a delay in treatment, or a failure to treat [2]. Technical errors resulting in surgical complications represent only about half of all events leading to a claim [2]. Furthermore, out of 258 medicolegal claims related to errors leading to surgical patient injuries, about 25% were attributed to a breakdown in communication before, during, or after surgery [3].

Thus, the surgical patient appears to be more at risk of sustaining an adverse outcome from clandestine system errors than by the imminent threat of the surgical blade "gone wrong". For example, a patient being operated at the wrong site is a "classical" system error much more than an individual human failure by a surgeon. Ten years ago, the American Academy of Orthopaedic Surgeons introduced a new standard of practice for surgeons to mark the body part to be operated on, in order to prevent wrong site operations. Meanwhile, the concept of a surgical "time-out" has been widely implemented in operating rooms throughout the United States as an improved method to verify patient identity, correct procedure, and intended-site operations [4,5]. Under these premises, the fact that the surgical "time-out" paradigm has not yet been implemented as a standard of care in most parts of the world, including Central Europe, appears incomprehensible and ethically unacceptable from a patient safety standpoint. Unfortunately, the "culture of blame" approach for dealing with individual surgical failures is still widely disseminated and has not yet been globally replaced by a formal and standardized measurement of process as a quality control tool in the management of surgical patients [6,7].

Despite the wide range of more than 200 official journals in the field of surgery, there is currently no single medical journal available which specializes on the issue of patient safety in surgery. In our opinion, this imbalance appears unjustified, since iatrogenic complications and system errors represent a significant contributing factor to morbidity and mortality after surgery. An evidence-based approach to quality improvement in surgical care must include the analysis of incidence and pattern of adverse events. This is particularly true for the analysis of "near-misses" (when an error was realized early enough to be aborted) and "no harm" events (when an error was not recognized in time to prevent it, but no adverse event occurred). Both scenarios bear the intrinsic risk of being neglected or trivialized, instead of being reported and reviewed as a "true" complication.

*Patient Safety in Surgery *(PSS) [8] is a peer-reviewed, open access, online journal which covers all aspects related to patient safety in surgery, including, but not limited to, surgical research (clinical and experimental), surgical technique, critical care, trauma management, perioperative safety aspects (anesthesia, etc), system issues, health care, nursing, political issues, as well as new products, new techniques, and teaching concepts related to the field.

The aim of this new journal is to increase the safety and quality of care for patients undergoing surgical procedures in all fields of surgery. The journal should complement traditional journals in surgery by filling an essential void, through providing a forum for discussion, analysis, and work-up of failures in the management of surgical patients. This scientific forum should lower the threshold for reporting adverse events in all fields of surgery with the long-term goal of increasing safety and quality of care for patients undergoing surgical procedures.

We wish to promote the reporting of errors, failures, and complications in the clinical management of surgical patients, while at the same time, we strive to protect the confidentiality of patients, authors, and health care specialists. For this reason, all reports which involve concrete individual cases, require a written consent from the patients or their legal guardians as well as from those health care professionals which were directly involved in patient care.

*Patient Safety in Surgery *follows a stringent, closed peer review process. Our renowned international Editorial Board [9] will ensure a high quality peer review by qualified experts in the field. Each submitted manuscript will first be screened by the Editors-in-Chief for suitability. All manuscripts deemed suitable for peer review will be assigned to at least two, usually three, expert referees. Authors will be requested to answer all referees' comments on a point-by-point analysis. The Editors-in-Chief will decide on whether to accept or reject a manuscript based on reviewer recommendations and their assessment of the manuscript. A first decision is aimed to be made within six weeks. Accepted articles will be published immediately online with their final citation on the day of acceptance, and will soon after be listed in PubMed.

*Patient Safety in Surgery *is published by BioMed Central (BMC) [10], an independent, open access publisher committed to ensuring that peer-reviewed biomedical research is freely available online without any charges, subscriptions, or other barriers. Recent reports have suggested that online open access journals represent the "future" in biomedical publishing, since researchers throughout the world will find and select relevant articles depending on their immediate and free availability over those with imposed financial or other barriers [11,12]. Open access availability was furthermore shown to correlate with the citation index and high impact factors once a new online journal has been established and tracked by the Institute for Scientific Information (ISI) for a defined period of time [12]. Finally, open access publishing is the future way to disseminate biomedical information in developing countries which cannot afford expensive institutional subscriptions to standard journals [13].

For this reason, *Patient Safety in Surgery *levies an article-processing charge (APC) for each accepted article [14]. In turn, the authors keep the full copyright on their published work, in contrast to traditional journals, which require a copyright release to the publisher. Authors from resource-poor countries may apply for an APC waiver, which is usually granted. Finally, all authors from institutions which are a BMC member organization will have the APC covered in full or in part by their institutional membership [10].

We believe that *Patient Safety in Surgery *will significantly impact the safety and quality of care for patients undergoing surgical procedures in all fields of surgery in the future. We urge you to consider submitting your next research article on the topic to our new journal.

## Competing interests

The author(s) declare that they have no competing interests.
